# ApoB/ApoA-1 Ratio as a Novel Prognostic Predictor in Patients With Primary Small Cell Carcinoma of the Esophagus

**DOI:** 10.3389/fonc.2020.00610

**Published:** 2020-04-24

**Authors:** Hao Chen, Ling-Yu Chu, Xiao-Hui Li, Yu-Hui Peng, Can-Tong Liu, Li-Ru Tian, Jian-Jun Xie, Yi-Wei Xu

**Affiliations:** ^1^State Key Laboratory of Oncology in South China, Collaborative Innovation Center for Cancer Medicine, Sun Yat-Sen University Cancer Center, Guangzhou, China; ^2^Department of Biochemistry and Molecular Biology, Shantou University Medical College, Shantou, China; ^3^Department of Clinical Laboratory Medicine, The Cancer Hospital of Shantou University Medical College, Shantou, China; ^4^Precision Medicine Research Center, Shantou University Medical College, Shantou, China; ^5^KingMed School of Laboratory Medicine, Guangzhou Medical University, Guangzhou, China

**Keywords:** ApoB/ApoA-1, Primary small cell carcinoma of the esophagus, prognosis, predictor, serum lipid

## Abstract

**Background and Aim:** Primary small cell carcinoma of the esophagus (SCCE) is a rarely aggressive disease characterized by rapid progression, widespread metastasis, and poor prognosis. This study was aimed to evaluate the prognostic significance of serum lipids for overall survival (OS) in SCCE patients.

**Methods:** We retrospectively analyzed SCCE patients in a training cohort (61 patients) and validated them in a validation cohort (27 patients). These cases were collected from Sun Yat-sen University Cancer Center from 2006 to 2017. Univariate and multivariate Cox survival analyses were performed to determine serum lipids as prognostic factors associated with the patient's OS. Time-dependent receiver operating characteristics (ROC) were used to compare predictive power of independent prognostic factors. The predictive accuracy and discriminative ability of the prognostic factors were measured by the concordance index (C-index) and decision curve, and were compared with the TNM stage system.

**Results:** On multivariate analysis of the training cohort, independent factors for survival were gender, BAR (ApoB/ApoA-1) and TNM stage. The area under the curve (AUC) of BAR+TNM stage in the training cohort was higher than that of TNM stage for OS, and similar result was observed in the validation cohort. The c-index of BAR+TNM stage for predicting the OS was 0.655 (95% CI = 0.571–0.740), which was higher than that of TNM stage [0.614 (95% CI = 0.530–0.698)] in the training cohort. In the validation cohort, the C-index of the BAR+TNM stage for predicting OS was also higher than that of the TNM stage [0.688 (95% CI: 0.570~0.806) vs. (0.512; 95% CI: 0.392~0.632)]. In addition, decision curve analysis also showed that the predictive accuracy of BAR+TNM stage for OS was higher than TNM stage both in the training and the validation cohorts.

**Conclusions:** BAR represents a promising prognostic indicator that might complement TNM stage in the prognosis of SCCE, and that warrant further assessment in large SCCE patient cohort.

## Introduction

Esophageal cancer is one of the most common malignant tumors in China, and its histological type is most common in squamous cell carcinoma (1). Primary small cell carcinoma of the esophagus (SCCE) is a relatively rare histopathological type, accounting for only 0.8–3.1% of all malignant tumors of the esophagus (2, 3). Since the British scholar Mckeown reported the first SCCE in 1952 (4), the United States estimates that the number of new cases of SCCE is about 130–395 per year, and the incidence is increasing year by year (5). As a high incidence country of esophageal cancer, increasing studies were focused on SCCE in China (1, 6). SCCE is a highly aggressive disease characterized by rapid progression, extensive metastasis and poor prognosis, with a higher incidence of males than females (7–10). At present, some studies have found a number of clinicopathological indicators that can assess the prognosis of SCCE, including lesions, TNM stage, VALSG stage, tumor inflammatory cell infiltration and treatment, but the results from some of these literatures are controversial (11–15). Therefore, it is necessary to explore new indicators that can evaluate the prognosis in order to improve the existing prognostic model and achieve a more accurate and comprehensive assessment of SCCE.

Lipid levels can reflect the state of lipid metabolism in the body, and many diseases are associated with disorders of dyslipidemia. Studies have shown that when tumors occur, serum lipids also change accordingly, and the changes in serum lipids in patients with different tumors are also diverse (16). Lipids are important components of cell membranes and metabolites of organisms, and play an important role in energy storage, structural composition and signal transduction (17, 18). Recently, a number of studies have demonstrated that serum lipid, such as total cholesterol (TC), triglyceride (TG), high-density lipoprotein cholesterol (HDL-C), low-density lipoprotein cholesterol (LDL-C), apolipoprotein A-1 (ApoA-1) and apolipoprotein B (ApoB) were associated with several types of cancer risk (18–21). Therefore, determining their content and ratio have important implications for the diagnosis and prognosis of cancer patients.

Although various studies have discussed the close relationship between serum lipids and cancers, studies of serum lipids and SCCE are very limited. The relationship between serum lipids and survival prognosis in patients with SCCE is also unclear. Therefore, in this study, we performed a retrospective study to assess the association of serum lipids with clinicopathological features and to predict the overall survival (OS) of SCCE in combination with TNM stage and serum lipids.

## Methods and Materials

### Study Population

In this study, we conducted a retrospective observational study. A total of 61 patients with SCCE were recruited as training cohort, which were obtained from the Sun Yat-sen University Cancer Center, from January 2006 to December 2016. A validation cohort was comprised of 27 patients with SCCE from the same center, from November 2010 to October 2017. We reviewed the detailed medical records of these patients who were diagnosed as SCCE based on barium swallow examination, spiral computed-tomography (CT) and endoscopic examination followed by histopathology. The histological criteria of small cell carcinoma of 2010 “WHO Pathology and Genetics of the Gastrointestinal System of the World Health Organization” was adopted. The pathological diagnosis of SCCE was determined in biopsy or resected specimens, and retrospective analysis was performed. Immunohistochemical staining was performed to determine the presence of common neuroendocrine markers including synaptophysin (Syn), neuron-specific enolase (NSE), chromogranin A (CgA), cytokeratin (CK), and lymphocyte antigen 56 (CD56). There were 55 and 26 patients underwent immunohistochemical examination in the training cohort and the validation cohort, respectively. The positive rate of each neuroendocrine markers is shown in Table S1.

Patients included in the analysis met the following criteria: (1) they were diagnosed as SCCE with histopathological examination; (2) they did not suffer from any cancer disease before SCCE diagnosis or receive any anti-cancer treatment; (3) they underwent chest CT examination, and the lung metastatic lesions could be ruled out; (4) they had complete follow-up data. The OS was defined as the interval between the initial diagnosis and either death of cancer or the last follow-up. The last follow-up was performed in November 2019. In this study, all serum results were obtained before treatment began. This study was approved by the Hospital Ethics Committee in Sun Yat-sen University Cancer Center in China and informed consents were obtained from all included participants. All work was complied with the principles of the Helsinki Declaration.

### Cut-Off Values of Prognostic Biomarkers

We collected clinicopathologic parameters of each patient as follows: gender, age, family history, tumor size, tumor location, treatment and pathologic TNM stage. The clinical stage of the disease was determined according to 8th edition of the AJCC TNM stage manual (22). The potential prognostic factors included APOA-1, APOB, HDL-C, LDL-C, TC, TG, BAR (BAR = ApoB/ApoA-1), BMI and PNI. PNI was calculated by the formula Alb (g/L) + 5 × lymphocyte count (×109/L). In this study, continuous variables were transformed into categorical variables. The best cut-off values for all variables were determined by X-tile (23) and were as follows: age (54 years), ApoA-1 (1.47 g/L), ApoB (0.97 g/L), HDL-C (0.97 mmol/L), LDL-C (2.81 mmol/L), TC (4.86 mmol/L), TG (1.10 g/L), BAR (0.72), BMI (20), and PNI (54).

### Statistical Analyses

Statistical analyses were performed using SPSS software, version 19.0 (SPSS Inc., Chicago, IL, USA) and R (version 3.4.4) for Windows. The Kaplan-Meier curves were used to calculate the survival rate, and the Log-rank test was used to compare them. Univariate analysis was performed to assess the importance of clinical and pathological features. Variables with a significant level of *P* ≤ 0.1 in univariate analysis were analyzed using multivariate Cox regression. A dynamic predictive nomogram model is built using all variables with a *P*-value of less than 0.05 in a multivariate model. The prognostic factors of the 1-, 3-, and 5-year OS were calibrated by comparing predicted survival with observed survival. The predictive accuracy and discriminative ability of the prognostic factors were measured by C-index and decision curve, and were compared with the TNM stage system. Hazard ratios (HR) and 95% confidence intervals (CI) were calculated using univariate and multivariate Cox proportional hazards regression models to assess the impacts of prognostic variable' OS. All statistical tests were two-sided, and *P* < 0.05 was considered statistically significant.

## Results

### Patient Characteristics

In training cohort, 61 patients met all criteria were enrolled in this study. The median age for these patients was 56 years (range 24–80 years), of which 44 (72.1%) were males and 17 (27.9%) were females. The numbers of patients of I-II, III and IV stage were 24 (39.3%), 22 (36.1%), and 15 (24.6%), respectively. At the time of the last follow-up, the median OS was 18 months. Similar results were observed in the validation cohort. Patient demographic and clinical characteristics are summarized in Table 1. In addition, there was no difference of BAR levels among patients undergoing surgery, surgery plus radiotherapy/chemotherapy, or radiotherapy plus chemoradiotherapy (Figure S1).

**Table 1:** Patient demographics and clinical characteristics.

	**Primary cohort**		**Validation cohort**	
**Characteristic**	**No**	**%**	**No**	**%**
Gender				
Male	44	72.1	21	77.8
Female	17	27.9	6	22.2
Age(years)				
<54	16	26.2	5	18.5
≥54	45	73.8	22	81.5
Family history				
Yes	13	21.3	7	25.9
No	48	78.7	20	74.1
Size(cm)				
<5	31	50.8	14	51.9
≥5	17	27.9	13	48.1
Unknown	13	21.3	0	0
TNM stage				
I-II	24	39.3	14	51.9
III	22	36.1	9	33.3
IV	15	24.6	4	14.8
Location				
Up	3	4.9	3	11.1
Middle	38	62.3	22	81.5
Low	20	32.8	2	7.4
Treatment				
Surgery	18	29.5	10	37
Surgery and Radiotherapy/Chemotherapy	18	29.5	10	37
Radiotherapy/Chemotherapy	25	41.0	7	25.9
ApoA-1 (g/L)				
<1.47	51	83.6	24	88.9
≥1.47	10	16.4	3	11.1
ApoB(g/L)				
<0.97	28	45.9	12	44.4
≥0.97	33	54.1	15	55.6
HDL-C(mmol/L)				
<0.97	17	27.9	4	14.8
≥0.97	44	72.1	23	85.2
LDL-C(mmol/L)				
<2.81	12	19.7	7	25.9
≥2.81	49	80.3	20	74.1
TC (mmol/L)				
<4.86	22	36.1	11	40.7
≥4.86	39	63.9	16	59.3
TG(g/L)				
<1.1	27	44.3	16	59.3
≥1.1	34	55.7	11	40.7
ApoB/ApoA-1				
<0.72	16	26.2	6	22.2
≥0.72	45	73.8	21	77.8
BMI				
<20	18	29.5	5	18.5
≥20	43	70.5	11	40.7
Unknown	0	0	11	40.7
PNI				
<54	32	52.5	18	66.7
≥54	29	47.5	9	33.3

*BMI, body mass index; PNI, prognostic nutritional index*.

### Univariate Analysis and Multivariate Cox Proportional Hazards Regression Analysis of the Overall Survival

**Figure 1:**
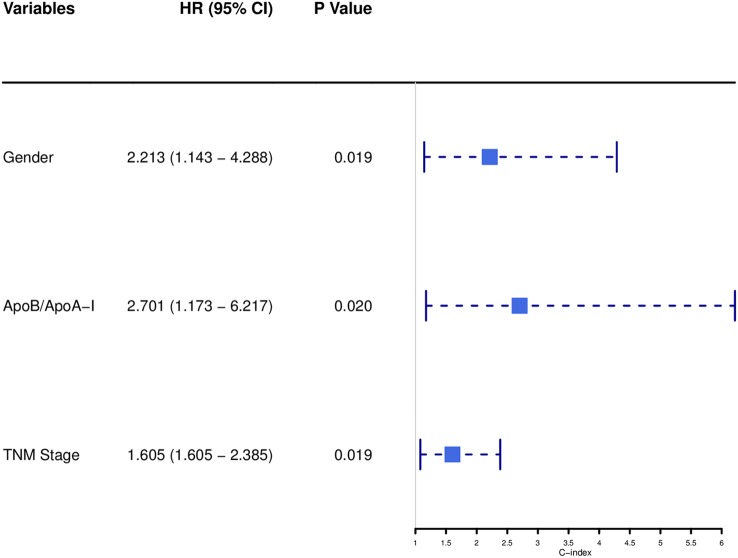
Forest plot showed the hazard ratio and 95% confidence interval for OS according to the Cox proportional hazards regression analysis in SCCE patients.

**Figure 2:**
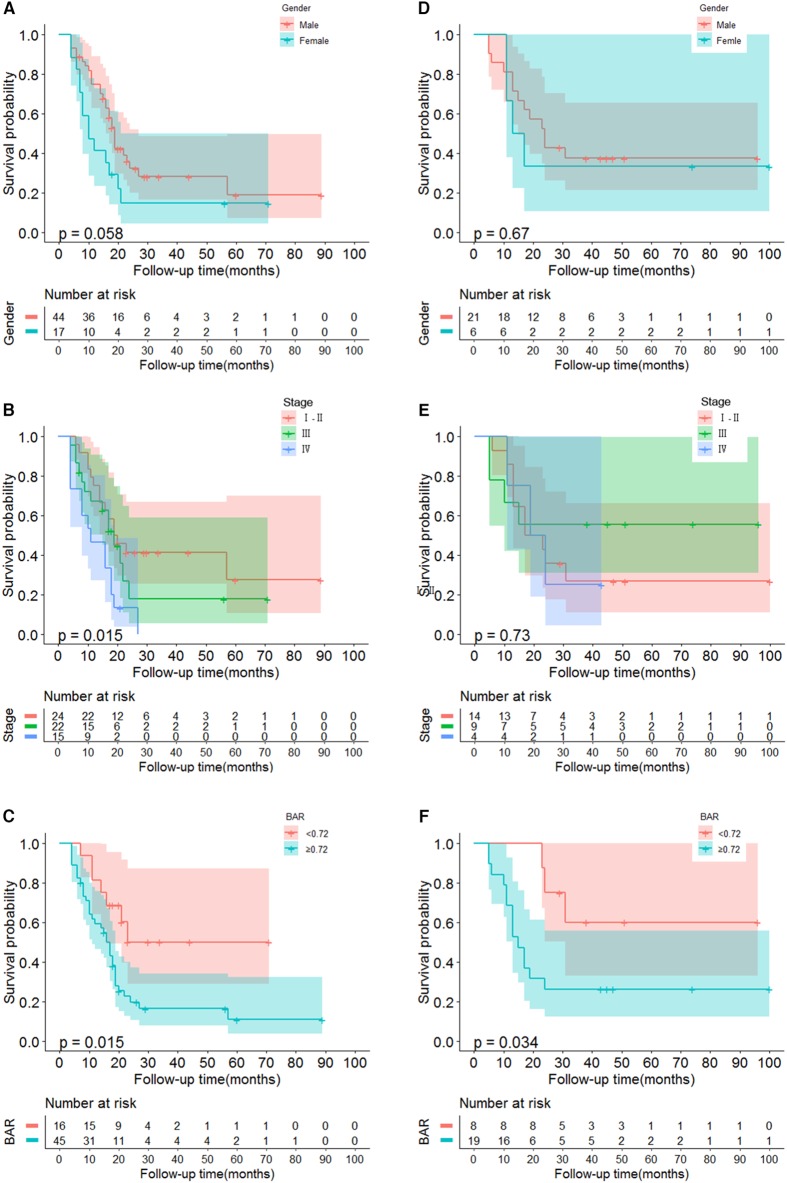
Kaplan-Meier curves for OS in SCCE patients. **(A–C)** The gender, TNM, BAR in SCCE patients in training cohort are plotted as a distribution. **(D–F)** The gender, TNM, BAR in SCCE patients in validation cohort are plotted as a distribution. BAR, ApoB/ApoA-1.

**Figure 3:**
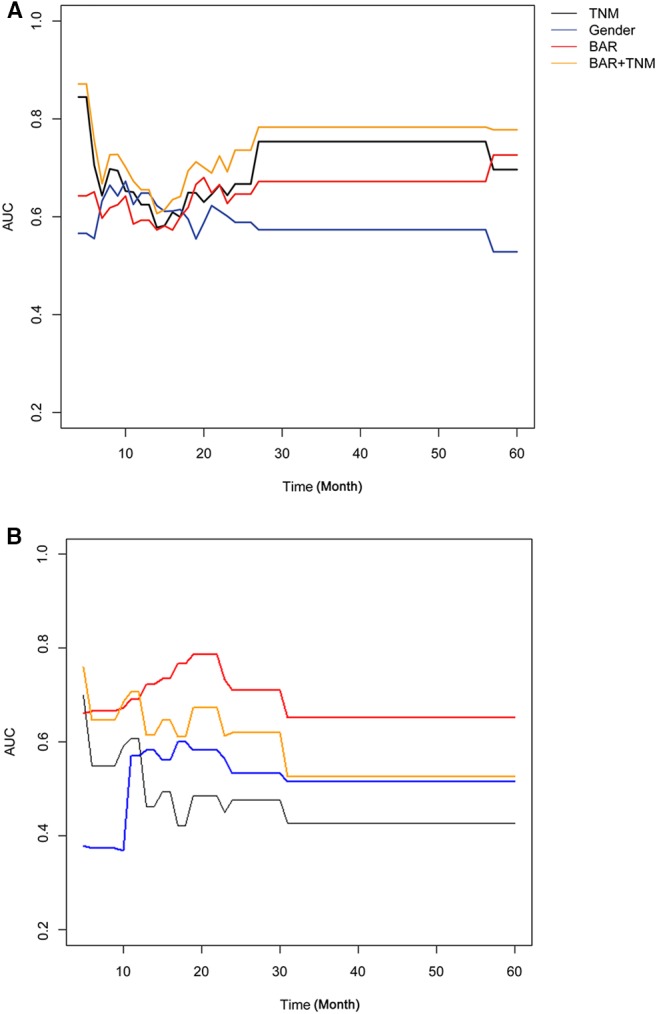
Time-dependent ROC curve for OS in SCCE patients in the training cohort **(A)** and validation cohort **(B)**. BAR, ApoB/ApoA-1.

Univariate analysis indicated that gender (*P* = 0.068), Tumor size (*P* = 0.026), TNM stage (*P* = 0.070), APOB (*P* = 0.077), LDL-C (*P* = 0.077), and BAR (*P* = 0.021) were associated with OS of SCCE patients. Then the prognostic factors significantly related to OS in univariate analysis were included in the multivariate Cox proportional risk regression analysis of OS. In multivariate analysis for OS with Cox regression, the results showed that the following variables remained independently prognostic: gender (*P* = 0.019, HR = 2.213; 95% CI: 1.14–4.29), TNM stage (*P* = 0.019, HR =1.605; 95% CI: 1.08–2.39) and BAR (*P* = 0.02, HR =2.701; 95% CI: 1.17–6.22). The detailed results of univariate and multivariate analyses are presented in Table 2. According to Cox proportional hazards regression analysis, the forest plot shows the hazard ratios and 95% confidence intervals for OS (Figure 1). The Kaplan-Meier curves for OS according to gender, TNM stage and BAR levels were significantly different, as confirmed by the log-rank test. As expected, we observed similar result for patients in the validation cohort with those in the training cohort (Figure 2). Moreover, the results of time-dependent ROC curve for OS showed that area under the curve (AUC) of BAR + TNM stage was higher than that of TNM stage, whether in the training cohort or the validation cohort (Figure 3).

**Table 2:** Univariate and multivariate Cox proportional hazards regression analysis for OS.

	**Univariate analysis**			**Multivariate analysis**		
	**HR**	**95% CI**	***P***	**HR**	**(95% CI)**	***P***
Gender						
Male	Reference			Reference		
Female	1.818	0.957–3.456	0.068	2.213	1.14–4.29	0.019
Age (years)						
<54	Reference					
≥54	1.450	0.693–3.306	0.324			
Family history						
Yes	Reference					
No	0.782	0.456–1.346	0.375			
Size(cm)						
<5	Reference					
≥5	0.442	0.215–0.909	0.026			
Unknown	0.464	0.195–1.103	0.082			
TNM stage						
I-II	Reference			1.605	1.08–2.39	0.019
III	0.357	0.196–1.054	0.070			
IV	0.513	0.244–1.165	0.079			
Location						
Up	Reference					
Middle	0.431	0.056–3.318	0.419			
Low	1.212	0.619–2.371	0.575			
Treatment						
Surgery	Reference					
Surgery and radiotherapy chemotherapy	1.090	0.541–2.199	0.809			
Radiotherapy/chemotherapy	0.950	0.431–2.097	0.900			
ApoA-1 (g/L)						
<1.47	Reference					
≥1.47	0.522	0.205–1.327	0.172			
ApoB (g/L)						
<0.97	Reference					
≥0.97	1.750	0.941–3.254	0.077			
HDL-C (mmol/L)						
<0.97	Reference					
≥0.97	1.608	0.790–3.273	0.190			
LDL-C (mmol/L)						
<2.81	Reference					
≥2.81	2.325	0.912–5.924	0.077			
TC (mmol/L)						
<4.86	Reference					
≥4.86	1.645	0.840–3.222	0.147			
TG (g/L)						
<1.1	Reference					
≥1.1	0.865	0.474–1.577	0.635			
ApoB/ApoA-1						
<0.72	Reference			2.701	1.17–6.22	0.020
≥0.72	0.385	0.171–0.868	0.021			
BMI						
<20	Reference					
≥20	0.724	0.375–1.098	0.336			
PNI						
<54	Reference					
≥54	0.630	0.343–1.159	0.137			

*HR, Hazard ratio; 95% CI, 95% confidence interval; BMI, body mass index; PNI, prognostic nutritional index*.

### Correlation Between BAR Levels and Treatment Outcome

To assess whether the BAR had an effect on the prognosis of different treatments, including surgery, surgery plus radiotherapy/chemotherapy, and radiotherapy plus chemoradiotherapy, we applied the same cutoff values mentioned above in all patients (total 88 samples both in the training and validation cohorts owing to the small sample size). They were divided into high- and low-expression groups. As shown in the Figure S2, the significant correlation between BAR expression and prognosis was only observed in patients receiving surgery plus radiotherapy/chemotherapy (*P* = 0.0041). Moreover, in the surgery plus radiotherapy/chemotherapy group, the high expression of BAR has a poor prognosis.

### The Nomogram for the Prediction of OS

**Figure 4:**
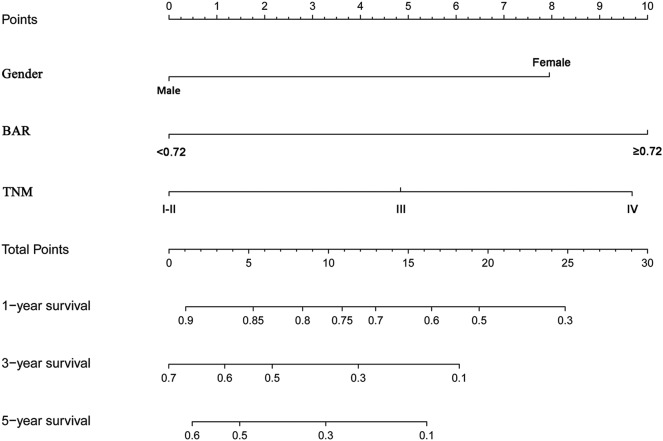
Nomogram model based on gender, BAR and TNM stage in the prediction of 1-, 3-, and 5- year OS in SCCE patients. The nomogram was used by summing the points identified on the points scale for each variable. The total points projected on the bottom scales indicate the probability of 1-, 3-, and 5-year survival. BAR, ApoB/ApoA-1.

To predict OS, a nomogram was established by multivariate Cox regression model according to significantly independent factors for OS. The models include gender, TNM stage and BAR (Figure 4). Each prognostic factor has a number of risk points, which can be obtained by drawing a vertical line directly upward from the corresponding value of the prognostic factor to an axis with a “point.” In order to determine the 1-, 3-, and 5-year OS probability of a specific patient from the “Total Points” which is the sum of the risk points, a vertical line can be drawn to the axis marked “1-, 3-, and 5-Year Overall Survival Probability.”

### Comparison of the Predictive Accuracy Between Prognostic Factors and Conventional Staging Systems

**Figure 5:**
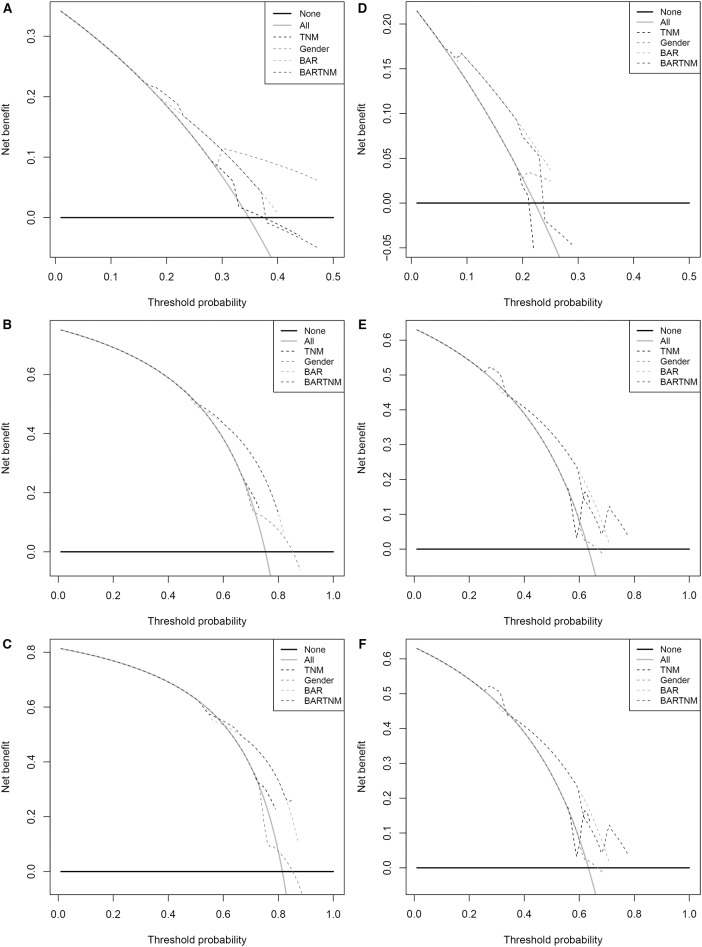
Decision curve analysis the predictive accuracy of BAR for OS in SCCE patients. **(A–C)** The decision curve of 1- **(A)**, 3- **(B)**, and 5- **(C)** year OS in training cohort; **(D–F)** The decision curve of 1-**(D)**, 3- **(E)**, and 5- **(F)** year OS in validation cohort. BAR, ApoB/ApoA-1.

Comparison of the predictive accuracy of prognostic factors and conventional staging systems were done using C-index and decision curve analysis for 1-, 3-, and 5-year survival. As shown in Table 3, although in the training cohort, there was no significant difference between gender, BAR, BAR+TNM stage and TNM stage systems (*P* > 0.05), the C-index of the BAR + TNM stage was better than that of the TNM stage systems alone [0.655 (95% CI = 0.571–0.740) vs. 0.614 (95% CI = 0.530–0.698)]. In the validation cohort, the C-index of the BAR+TNM stage in predicting OS was 0.688 (95% CI: 0.570~0.806), which was higher than that of the TNM system (0.512; 95% CI: 0.392~0.632). In addition, the analysis of the decision curves for 1-, 3-, and 5-year survival rates shows that the current BAR+TNM stage systems seems to have higher prediction accuracy than the TNM stage systems in training cohort. This result was verified in the validation cohort (Figure 5). Due to the small sample size in this study, we combined training and validation cohorts and used net reclassification improvement (IDI) and integrated discrimination improvement (NRI) to evaluate the accuracy of BAR + TNM stage prediction of survival. In Table 4, the NRI suggested that the predictive accuracy of BAR+TNM stage was better than that of the TNM stage system. Furthermore, compared to the TNM stage, IDI shows that the accuracy of the BAR + TNM stage for predicting 1-, 3-, and 5-year OS was improved. To conclude, BAR + TNM stage had better net benefit and predictive accuracy than those of the TNM stage alone.

**Table 3:** The C-index of gender, BAR, TNM stage and BAR+TNM stage for prediction of OS in the SCCE.

	**Training cohort**		**Validation cohort**	
**Factors**	**C-index (95% CI)**	***P***	**C-index (95% CI)**	***P***
For OS				
Gender	0.583(0.507~0.658)		0.529 (0.420~0.636)	
BAR	0.592(0.522~0.661)		0.658 (0.550~0.747)	
TNM stage	0.614(0.530~0.698)		0.512 (0.392~0.632)	
BAR + TNM stage	0.655(0.571~0.740)		0.688 (0.570~0.806)	
Gender vs. TNM stage		0.541		0.400
BAR vs. TNM stage		0.595		0.006
BAR + TNM stage vs. TNM stage		0.151		0.002

*C-index, concordance index; CI, confidence interval; BAR, APOB/APOA-1; P-values are calculated based on normal approximation using function rcorrp.cens in Hmisc package*.

**Table 4:** A comparison of discriminatory ability of gender, BAR and BAR +TNM stage with TNM stage using NRI and IDI.

	**1-Year**				**3-Year**				**5-Year**			
	**NRI**	***P***	**IDI**	***P***	**NRI**	***P***	**IDI**	***P***	**NRI**	***P***	**IDI**	***P***
Gender vs. TNM stage	6.9%	0.593	1.9%	0.521	7.6%	0.637	−0.1%	0.989	−15.1%	0.450	−5.1%	0.248
BAR vs. TNM stage	19.0%	0.160	2.8%	0.300	20.0%	0.553	6.5%	0.230	14.4%	0.895	3.6%	0.771
BAR+TNM stage vs.	19.0%	0.060	3.1%	0.108	30.0%	0.188	6.5%	0.132	14.4%	0.633	3.8%	0.723
TNM stage												

*NRI, Net Reclassification Improvement; IDI, Integrated Discrimination Improvement*.

## Discussion

In recent years, with the rising incidence of SCCE, increasing studies have focused on SCCE (1, 5, 6). Although tumor stage based on the American Joint Committee on Esophageal Cancer (AJCC) is the primary basis for judging the prognosis of SCCE (24), even at the same stage of SCCE, there is a large difference in OS. The current stage system is based entirely on the anatomical extent of the disease. However, stage systems do not fully reflect the biological heterogeneity of SCCE patients, and other risk factors are not considered in current stage systems. Thus, the current TNM stage system is not sufficient for prognosis of SCCE. It is necessary to find effective and reliable prognostic factors to predict SCCE prognosis and identify individuals with poor prognosis.

In this study, we used univariate analysis and subsequent multivariate analysis to determine gender, BAR, and TNM stage as independent prognostic factors for SCCE patients. In line with the previous studies, TNM stage was significant prognostic markers for resected SCCE patients (24). The age factor does not reflect the impact on survival, which may be related to the high degree of malignancy of SCCE. Next, a nomogram for predicting survival was developed and these three variables were incorporated into the nomogram. There was no statistically significant difference in *P*-values between the nomogram and the TNM stage, which may be the reason for the limited samples of SCCE. But the C-index of the BAR+TNM stage predicted OS with an accuracy of 0.655, which showed better prediction of OS than the TNM stage system (0.614). Similar results were observed in the validation cohort. At the same time, the decision curve analysis of 1-, 3-, and 5-year survival rates showed that the BAR+TNM stage prediction model had greater clinical application potential than the TNM stage system. Therefore, BAR+TNM stage seems to be more suitable as a prognostic factor for SCEE than TNM stage alone. To our knowledge, this study is the first retrospective analysis of the prognostic role of pretreatment of serum lipids in SCCE. Here, SCCE patients were divided into two risk groups by BAR ratio, and the results showed that when BAR ≥ 0.72 the prognosis was poor. Previous literature has also reported that ApoB/ApoA-1 is an independent prognostic factor for gastric cancer (25). In addition, the BAR assay is relatively inexpensive and routinely performed during preoperative examinations. Therefore, the BAR+TNM stage may be a reliable tool for predicting survival in patients with SCCE and contribute to individualized treatment decisions.

Apolipoprotein is a protein part of plasma lipoprotein, a protein that binds and transports serum lipids to various tissues of the body for metabolism and utilization. ApoA-1-encoded apolipoprotein A-1 (ApoA-1) is a major protein component of high-density lipoprotein (HDL) and plays an important role in the neutralization and clearance of lipopolysaccharide (26), reversible transport of cholesterol (27), inhibition Inflammatory response caused by toxins (28). ApoB is a ligand for low-density lipoprotein (LDL) receptors, which cleans up low-density lipoproteins in the body, participates in the synthesis and secretion of very low-density lipoprotein (VLDL), transports fat and cholesterol (29). Considering the imbalance of lipid metabolism in cancer patients, indicators containing multiple lipids can more accurately and comprehensively reflect changes in serum lipids. A study reports that LDL-C and HDL-C ratios (LHR) are prognostic factors for colorectal cancer patients, providing more prognostic information than single LDL-C or HDL-C (30). In addition, Mazidi et al. studies indicated that the ApoB/ApoA-I ratio might be a useful predictor of the risk for cancer mortality (31). It was also shown that this ratio may even better predict cancer risk than inflammatory markers and lipid biomarkers (31). Therefore, we explored the relationship between ApoB / ApoA-1(BAR) and SCCE prognosis in our study and found that BAR is an independent prognostic factor for SCCE. These findings suggest that the measurement of Apos may have important clinical significance in identifying at-risk populations with fatal cancer disease. And in our study, the prognosis was poor when the BAR ratio was increased. This may be related to a decrease in ApoA-1 levels, as low concentrations of ApoA-1 are closely associated with the development, progression, and prognosis of multiple malignancies (18–21, 31). While ApoB's research focuses on cardiac metabolic disorders, the study found that individuals with higher ApoB levels might have a greater cancer mortality risk (31, 32). Several possible mechanisms can be used to explain the prognostic value of ApoB/ApoA-1 for SCCE. First, studies have shown that low ApoA-1 concentrations inhibit tumor progression through its anti-inflammatory effects (33). ApoA-1 may play an anti-inflammatory role mainly through the binding ability between macrophages and cell phospholipids (33, 34). Lysophosphatidylcholines, such as lysophosphatidic acid (LPA), are the proliferation activators of many tumors, and ApoA-1 can bind with LPA to inhibit the formation of tumors induced by LPA (35). In addition, there is an interaction between ApoA-1 and the body's inflammatory response, which reduces the rate of liver synthesis and secretion of ApoA-1, and serum low ApoA-1 concentration can indirectly lead to increased cytokine release and strong inflammatory response against tumor cells (20, 34, 36). Second, serum lipids inhibit the growth and metastasis of tumor cells by stabilizing the stability of prostaglandin 2 (PGI2) (37). Third, ApoA-1, a potential immunomodulator, converts the tumor-associated macrophage phenotype from a tumor-promoting phenotype (M2 type) to an anti-tumor phenotype (M1 type) (38, 39). Fourth, ApoA-1 inhibits tumor angiogenesis by other means to inhibit tumor growth, but its specific mechanism is not fully understood (40, 41). Fifth, ApoA-1 may participate in the development of tumors by regulating the cholesterol level of cells and participating in the lipid metabolism of cells (42, 43).

Although BAR+TNM stage might be used as a useful tool for clinicians to select and plan treatment strategies for SCCE patients, our research has several limitations. First, our study is a retrospective study with possible bias in the retrospective data collection process. However, because of the extremely low incidence of SCCE, one could envision that it is really hard to conduct a prospective study to evaluate the prognostic value of BAR+TNM stage. What needs to be pointed out is that our results were verified in an independent cohort. Thus, we believe our retrospective study still offers potential application value. Second, since the database used to generate the prognostic factors just consists of patient data from a single cancer center, it is necessary to obtain larger samples from other research institutions to validate the results.

In summary, our findings indicate that convenient serological indicator (i.e., BAR) combined with TNM stage seems to be more accurate in predicting OS than the traditional TNM stage system. It is appealing to imagine that in the SCCE setting, this serum lipid marker could be utilized for disease monitoring and prognostic prediction. In the near future, a large-scale, multicenter validation study is warranted to address the relationship between BAR and SCCE prognosis, and whether this serum markers BAR for the prognosis of SCCE could achieve real clinical benefit needs further verification.

## Data Availability Statement

All datasets generated for this study are included in the article/Supplementary Material.

## Ethics Statement

The studies involving human participants were reviewed and approved by Hospital Ethics Committee in Sun Yat-sen University Cancer Center. The patients/participants provided their written informed consent to participate in this study.

## Author Contributions

HC, L-YC, and X-HL designed the study, searched the literature, collected patient samples and clinical data, analyzed and interpreted the data, and wrote the manuscript. Y-HP, C-TL, and L-RT analyzed and interpreted the data. J-JX designed the study and revised the paper. Y-WX conceptualized and designed the study, supervised the project, and revised the paper. All authors read and approved the final manuscript.

## Conflict of Interest

The authors declare that the research was conducted in the absence of any commercial or financial relationships that could be construed as a potential conflict of interest.

## Abbreviations

SCCE: primary small cell carcinoma of the esophagus
OS: overall survival
ROC: receiver operating characteristics
C-index: concordance index
ApoA-1: apolipoprotein A-1
ApoB: apolipoprotein B
BAR: ApoB/ApoA-1 ratios
AUC: area under curves
TC: total cholesterol
TG: triglyceride
HDL-C: high-density lipoprotein cholesterol
LDL-C: low-density lipoprotein cholesterol
CT: computed-tomography
Syn: synaptophysin
NSE: neuron-specific enolase
CgA: chromogranin A
CK: cytokeratin
CD56: lymphocyte antigen 56
BMI: body mass index
PNI: prognostic nutritional index
HR: hazard ratios
CI: 95% confidence intervals
NRI: net reclassification improvement
IDI: integrated discrimination improvement
HDL: high-density lipoprotein
LDL: low-density lipoprotein
VLDL: very low-density lipoprotein
LHR: LDL-C/HDL-C ratios
LPA: lysophosphatidic acid
PGI2: prostaglandin 2.

## References

[1] Bray F Ferlay J Soerjomataram I Siegel RL Torre LA Jemal A. Global cancer statistics 2018: GLOBOCAN estimates of incidence and mortality worldwide for 36 cancers in 185 countries. CA Cancer J Clin. (2018) 68:394–424. 10.3322/caac.21492 30207593

[2] Xu X Yang Y Cao L Li F Zhao J Guo B. Lymph node metastasis and recurrence in primary small cell Carcinoma of the esophagus: a retrospective study of 125 cases. Cancer Biother Radiopharm. (2019) 34:459–63. 10.1089/cbr.2019.2800 31120315

[3] Chen WW Wang F Zhang DS Luo HY Wang ZQ Wang FH. Primary small cell carcinoma of the esophagus: clinicopathological study of 44 cases. BMC Cancer. (2014) 14:222. 10.1186/1471-2407-14-222 24666414 PMC3987173

[4] McKeown F. Oat-cell carcinoma of the oesophagus. J Pathol Bacteriol. (1952) 64:889–91. 10.1002/path.1700640420 13000600

[5] Walenkamp AM Sonke GS Sleijfer DT. Clinical and therapeutic aspects of extrapulmonary small cell carcinoma. Cancer Treat Rev. (2009) 35:228–36. 10.1016/j.ctrv.2008.10.007 19068273

[6] Yekeler E Koca T Vural S. A rare cause of the cough: primary small cell carcinoma of esophagus-case report. Case Rep Med. (2012) 2012:870783. 10.1155/2012/870783 22461794 PMC3296277

[7] Pantvaidya GH Pramesh CS Deshpande MS Jambhekar NA Sharma S Deshpande RK. Small cell carcinoma of the esophagus: the Tata Memorial Hospital experience. Ann Thorac Surg. (2002) 74:1924–7. 10.1016/S0003-4975(02)04061-4 12643374

[8] Nishimaki T Suzuki T Nakagawa S Watanabe K Aizawa K Hatakeyama K. Tumor spread and outcome of treatment in primary esophageal small cell carcinoma. J Surg Oncol. (1997) 64:130–4. 10.1002/(sici)1096-9098(199702)64:2<130::aid-jso8>3.0.co;2-c 9047250

[9] Hosokawa A Shimada Y Matsumura Y Yamada Y Muro K Hamaguchi T. Small cell carcinoma of the esophagus. Analysis of 14 cases and literature review. Hepatogastroenterology. (2005) 52:1738–41. 16334769

[10] Nayal B Vasudevan G Rao AC Kudva R Valliathan M Mathew M. Primary small cell Carcinoma of the esophagus - an eight year retrospective study. J Clin Diagn Res. (2015) 9:EC04–6. 10.7860/JCDR/2015/12464.5927 26155481 PMC4484073

[11] Deng HY Ni PZ Wang YC Wang WP Chen LQ. Neuroendocrine carcinoma of the esophagus: clinical characteristics and prognostic evaluation of 49 cases with surgical resection. J Thorac Dis. (2016) 8:1250–6. 10.21037/jtd.2016.04.21 27293844 PMC4886019

[12] Rice TW Rusch VW Ishwaran H Blackstone EH Worldwide Esophageal Cancer C Cancer of the esophagus and esophagogastric junction: data-driven stage for the seventh edition of the American Joint Committee on Cancer/International Union against Cancer stage manuals. Cancer. (2010) 116:3763–73. 10.1002/cncr.25146 20564099

[13] Lu JM Liang J Wang JW He J Xiao ZF Zhang HX. [Clinical analysis of 126 patients with primary small cell carcinoma of the esophagus]. Zhonghua Zhong Liu Za Zhi. (2009) 31:121–5. 10.3760/cma.j.issn.0253-3766.2009.02.010 19538888

[14] Zhang Y Ren H Wang L Ning Z Zhuang Y Gan J. Clinical impact of tumor-infiltrating inflammatory cells in primary small cell esophageal carcinoma. Int J Mol Sci. (2014) 15:9718–34. 10.3390/ijms15069718 24886814 PMC4100116

[15] Chen WW Wang F Chen S Wang L Ren C Luo HY. Detailed analysis of prognostic factors in primary esophageal small cell carcinoma. Ann Thorac Surg. (2014) 97:1975–81. 10.1016/j.athoracsur.2014.02.037 24726599

[16] Ganjali S Ricciuti B Pirro M Butler AE Atkin SL Banach M. High-density lipoprotein components and functionality in cancer: state-of-the-Art. Trends Endocrinol Metab. (2019) 30:12–24. 10.1016/j.tem.2018.10.004 30473465

[17] Luo X Cheng C Tan Z Li N Tang M Yang L. Emerging roles of lipid metabolism in cancer metastasis. Mol Cancer. (2017) 16:76. 10.1186/s12943-017-0646-3 28399876 PMC5387196

[18] Beloribi-Djefaflia S Vasseur S Guillaumond F. Lipid metabolic reprogramming in cancer cells. Oncogenesis. (2016) 5:e189. 10.1038/oncsis.2015.49 26807644 PMC4728678

[19] Ceres K Fitzgerald H Quiznon KS McDonough S Behling-Kelly E. Immunohistochemical labeling of low-density lipoprotein receptor and scavenger receptor Class B type 1 are increased in Canine lymphoma. Front Vet Sci. (2018) 5:340. 10.3389/fvets.2018.00340 30687727 PMC6336922

[20] Georgila K Vyrla D Drakos E. Apolipoprotein A-I (ApoA-I), immunity, inflammation and cancer. Cancers. (2019) 11:1097. 10.3390/cancers11081097 31374929 PMC6721368

[21] Lee G Jeong YS Kim DW Kwak MJ Koh J Joo EW. Clinical significance of APOB inactivation in hepatocellular carcinoma. Exp Mol Med. (2018) 50:1–12. 10.1038/s12276-018-0174-2 30429453 PMC6235894

[22] Edge SB Compton CC The American Joint Committee on Cancer: the 7th edition of the AJCC cancer stage manual and the future of TNM. Ann Surg Oncol. (2010) 17:1471–4. 10.1245/s10434-010-0985-4 20180029

[23] Camp RL Dolled-Filhart M Rimm DL. X-tile: a new bio-informatics tool for biomarker assessment and outcome-based cut-point optimization. Clin Cancer Res. (2004) 10:7252–9. 10.1158/1078-0432.CCR-04-0713 15534099

[24] Wang SY Mao WM Du XH Xu YP Zhang SZ The 2002 AJCC TNM classification is a better predictor of primary small cell esophageal carcinoma outcome than the VALSG stage system. Chin J Cancer. (2013) 32:342–52. 10.5732/cjc.012.10161 23114087 PMC3845624

[25] Ma MZ Yuan SQ Chen YM Zhou ZW. Preoperative apolipoprotein B/apolipoprotein A1 ratio: a novel prognostic factor for gastric cancer. Onco Targets Ther. (2018) 11:2169–76. 10.2147/OTT.S156690 29713185 PMC5907890

[26] Guo L Ai J Zheng Z Howatt DA Daugherty A Huang B. High density lipoprotein protects against polymicrobe-induced sepsis in mice. J Biol Chem. (2013) 288:17947–53. 10.1074/jbc.M112.442699 23658016 PMC3689940

[27] van der Vorst EPC. High-density Lipoproteins and Apolipoprotein A1. Subcell Biochem. (2020) 94:399–420. 10.1007/978-3-030-41769-7_16 32189309

[28] Ahmed HM Miller M Nasir K McEvoy JW Herrington D Blumenthal RS. Primary low level of high-density lipoprotein cholesterol and risks of coronary heart disease, Cardiovascular disease, and death: results from the multi-ethnic study of Atherosclerosis. Am J Epidemiol. (2016) 183:875–83. 10.1093/aje/kwv305 27189327 PMC4867155

[29] Morita SY. Metabolism and modification of Apolipoprotein B-containing lipoproteins involved in Dyslipidemia and Atherosclerosis. Biol Pharm Bull. (2016) 39:1–24. 10.1248/bpb.b15-00716 26725424

[30] Liao F He W Jiang C Yin C Guo G Chen X. A high LDL-C to HDL-C ratio predicts poor prognosis for initially metastatic colorectal cancer patients with elevations in LDL-C. Onco Targets Ther. (2015) 8:3135–42. 10.2147/OTT.S90479 26604782 PMC4629979

[31] Mazidi M Katsiki N Mikhailidis DP Radenkovic D Pella D Banach M. Apolipoprotein B/Apolipoprotein A-I ratio is a better predictor of cancer mortality compared with C-reactive protein: results from two multi-ethnic US populations. J Clin Med. (2020) 9:E170. 10.3390/jcm9010170 31936330 PMC7019626

[32] Atchison EA Gridley G Carreon JD Leitzmann MF McGlynn KA Risk of cancer in a large cohort of US veterans with diabetes. Int J Cancer. (2011) 128:635–43. 10.1002/ijc.25362 20473855 PMC2962873

[33] Penson PE Long DL Howard G Toth PP Muntner P Howard VJ. Associations between very low concentrations of low density lipoprotein cholesterol, high sensitivity C-reactive protein, and health outcomes in the reasons for geographical and racial differences in stroke (REGARDS) study. Eur Heart J. (2018) 39:3641–53. 10.1093/eurheartj/ehy533 30165636 PMC6195947

[34] Sathiyakumar V Kapoor K Jones SR Banach M Martin SS Toth PP. Novel therapeutic targets for managing Dyslipidemia. Trends Pharmacol Sci. (2018) 39:733–47. 10.1016/j.tips.2018.06.001 29970260

[35] Su F Kozak KR Imaizumi S Gao F Amneus MW Grijalva V. Apolipoprotein A-I (apoA-I) and apoA-I mimetic peptides inhibit tumor development in a mouse model of ovarian cancer. Proc Natl Acad Sci USA. (2010) 107:1997–2002. 10.1073/pnas.1009010107 21041624 PMC2993420

[36] Camont L Chapman MJ Kontush A. Biological activities of HDL subpopulations and their relevance to cardiovascular disease. Trends Mol Med. (2011) 17:594–603. 10.1016/j.molmed.2011.05.013 21839683

[37] Feng W Gao X McClung G Zhu S Ishihara K Brash JL. Methacrylate polymer layers bearing poly(ethylene oxide) and phosphorylcholine side chains as non-fouling surfaces: in vitro interactions with plasma proteins and platelets. Acta Biomater. (2011) 7:3692–9. 10.1016/j.actbio.2011.06.007 21693202

[38] Zamanian-Daryoush M Lindner D Tallant TC Wang Z Buffa J Klipfell E. The cardioprotective protein apolipoprotein A1 promotes potent anti-tumorigenic effects. J Biol Chem. (2013) 288:21237–52. 10.1074/jbc.M113.468967 23720750 PMC3774392

[39] Zamanian-Daryoush M DiDonato JA. Apolipoprotein A-I and cancer. Front Pharmacol. (2015) 6:265. 10.3389/fphar.2015.00265 26617517 PMC4642354

[40] Tan JT Ng MK Bursill CA. The role of high-density lipoproteins in the regulation of angiogenesis. Cardiovasc Res. (2015) 106:184–93. 10.1093/cvr/cvv104 25759067

[41] Gao F Vasquez SX Su F Roberts S Shah N Grijalva V. L-5F, an apolipoprotein A-I mimetic, inhibits tumor angiogenesis by suppressing VEGF/basic FGF signaling pathways. Integr Biol (Camb). (2011) 3:479–89. 10.1039/c0ib00147c 21283904 PMC3248743

[42] Smith B Land H. Anticancer activity of the cholesterol exporter ABCA1 gene. Cell Rep. (2012) 2:580–90. 10.1016/j.celrep.2012.08.011 22981231 PMC3462268

[43] Mendivil CO Furtado J Morton AM Wang L Sacks FM. Novel pathways of Apolipoprotein A-I metabolism in high-density lipoprotein of different sizes in humans. Arterioscler Thromb Vasc Biol. (2016) 36:156–65. 10.1161/ATVBAHA.115.306138 26543096 PMC4690755

