# Supplementary material for: ApoB/ApoA-1 Ratio as a Novel Prognostic Predictor in Patients With Primary Small Cell Carcinoma of the Esophagus
Source: Front Oncol. 2020 Apr 24;10:610. doi: 10.3389/fonc.2020.00610 (PMC7193088; doi:10.3389/fonc.2020.00610)
Supplement: Supplementary file 1 [file Data_Sheet_1.DOCX]

**Supplemental material**

**ApoB/ApoA-1 Ratio as a Novel Prognostic Predictor in Patients with Primary Small Cell Carcinoma of the Esophagus**

**Supplementary Table S1**

| Table S1. Positive rate of neuroendocrine markers in SCCE patients | | | | | |
| --- | --- | --- | --- | --- | --- |
| Neuroendocrine markers | Training cohort | |  | Validation cohort | |
|  | N | Positive (%) |  | N | Positive (%) |
| synaptophysin (Syn) | 53 | 50(94.3) |  | 26 | 24(92.3) |
| neuron-specific enolase (NSE) | 44 | 37(77.3) |  | 22 | 14(63.3) |
| chromogranin A (CgA) | 54 | 28(51.9) |  | 26 | 19(73.1) |
| cytokeratin (CK) | 48 | 45(93.8) |  | 20 | 18(90.0) |
| lymphocyte antigen 56 (CD56) | 52 | 49(94.2) |  | 25 | 24(96.0) |

N: indicates the number of the SCCE patients undergoing related marker detection.

**Supplementary Figure S1**

**
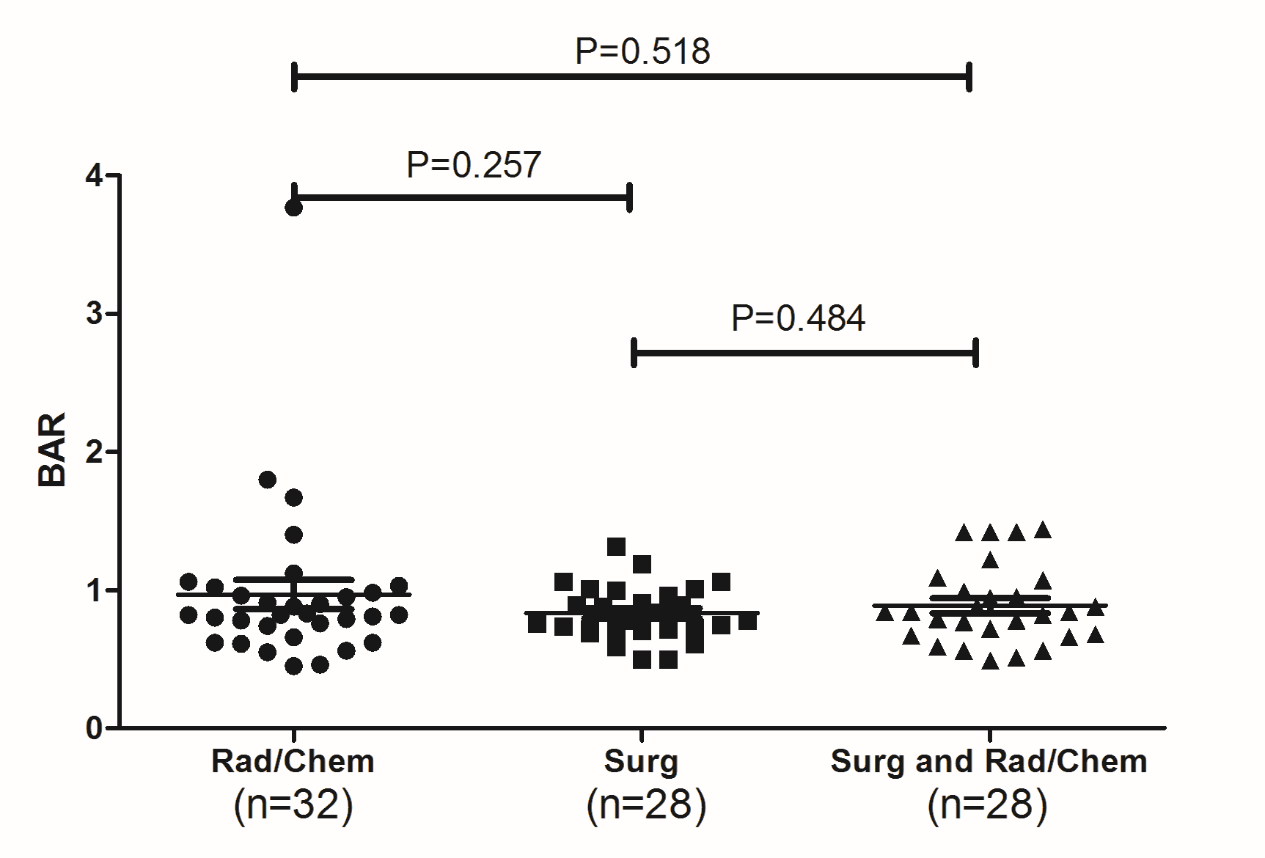
**

**Figure S1. Expression level of bar in rad/chem group, surg group,** **surg and rad/chem group.** BAR: ApoB/ApoA-1; Rad: radiotherapy; Chem: chemotherapy; Surg: surgery.

**Supplementary Figure S2**


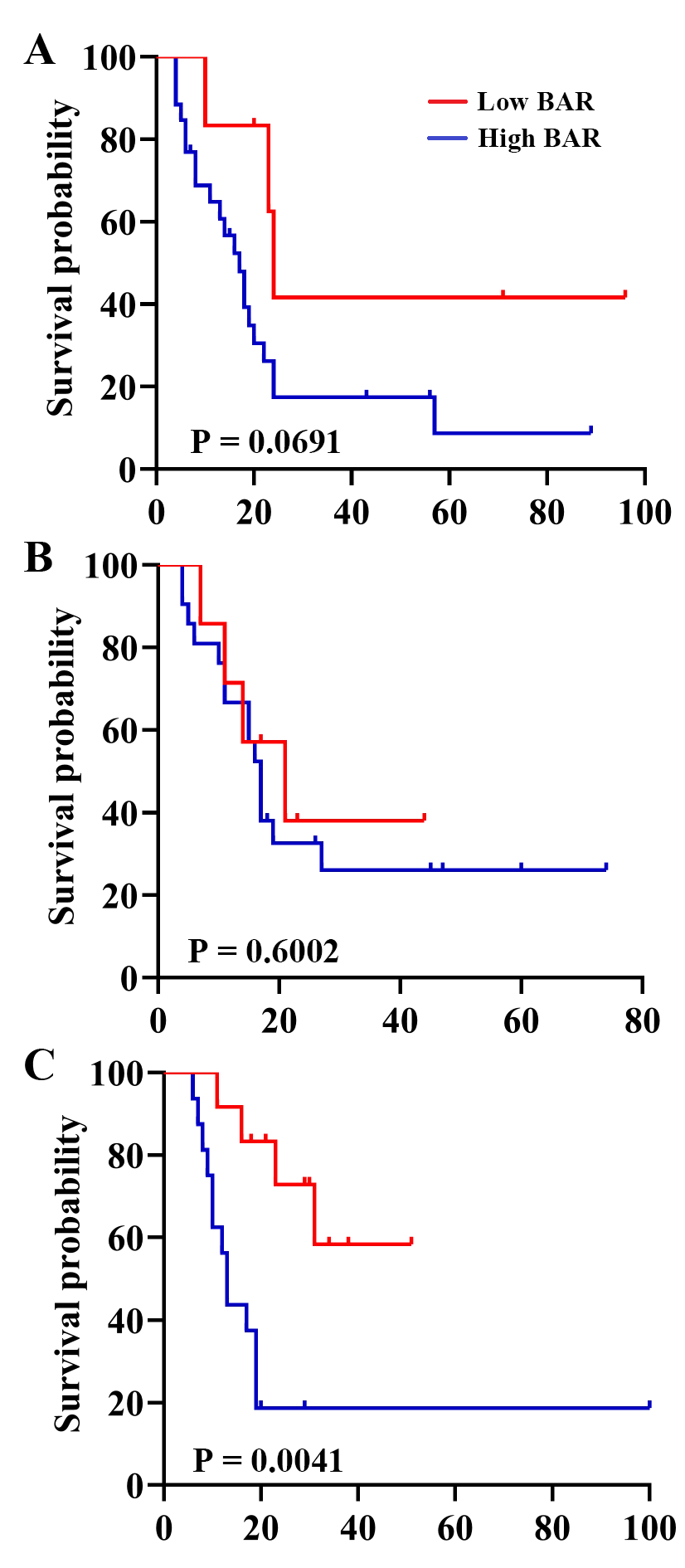


**Figure S2. Kaplan-Meier curves for OS in different treatments.** A. Rad/Chem group; B. Surgery group; C. Surgery and Rad/Chem group. Rad: radiotherapy; Chem: chemotherapy. P values were calculated using log-rank test.
